# A rat model of cirrhosis with well-differentiated hepatocellular carcinoma induced by thioacetamide

**DOI:** 10.3389/fgstr.2024.1427820

**Published:** 2024-11-12

**Authors:** Zhiping Hu, Takeshi Kurihara, Yiyue Sun, Zeliha Cetin, Rodrigo M. Florentino, Lanuza A. P. Faccioli, Zhenghao Liu, Bo Yang, Alina Ostrowska, Joseph D. Locker, Alejandro Soto-Gutierrez, Evan R. Delgado

**Affiliations:** ^1^ Department of Pathology, University of Pittsburgh, Pittsburgh, PA, United States; ^2^ Department of Pathology, Center for Transcriptional Medicine, University of Pittsburgh, Pittsburgh, PA, United States; ^3^ Department of Pathology, Pittsburgh Liver Research Center, University of Pittsburgh, Pittsburgh, PA, United States

**Keywords:** thioacetamide, cholangiocarcinoma (CCA), hepatocellular carcinoma, fibrosis, cirrhosis, mixed phenotype

## Abstract

Hepatocellular carcinoma (HCC) is a leading cause of cancer-related deaths, and commonly associated with hepatic fibrosis or cirrhosis. This study aims to establish a rat model mimicking the progression from liver fibrosis to cirrhosis and subsequently to HCC using thioacetamide (TAA). We utilized male Lewis rats, treating them with intra-peritoneal injections of TAA. These rats received bi-weekly injections of either 200 mg/kg TAA or saline (as a control) over a period of 34 weeks. The development of cirrhosis and hepatocarcinogenesis was monitored through histopathological examinations, biochemical markers, and immunohistochemical analyses. Our results demonstrated that chronic TAA administration induced cirrhosis aggressive cholangiocarcinoma in addition to well-differentiated HCC, providing a model for early-stage, stage and a mixed liver cancer phenotype. This model is characterized by increased fibrosis, altered liver architecture, and increased hepatocyte proliferation. Biochemical analyses revealed significant alterations in liver function markers, including elevated alpha-fetoprotein (AFP) levels, without affecting kidney function or causing significant weight loss or mortality in rats. This TAA-induced cirrhosis and mixed HCC rat model successfully replicates the clinical progression of human HCC, particularly in terms of liver function impairment and early-stage liver cancer characteristics. It serves as a valuable tool for future research on the mechanisms of antitumor drugs in tumor initiation and development.

## Introduction

Liver cancer stands as the third most common cause of death from cancer, with hepatocellular carcinoma (HCC) being the primary type responsible for this high mortality rate (1). Remarkably, about 90% of HCC cases are closely associated with liver fibrosis or cirrhosis, both of which are outcomes of chronic liver damage. Although various carcinogenic pathways may be involved in these conditions, the transformation of liver tissue into a malignant state is significantly influenced by liver fibrosis or cirrhosis (2). This transformation process impacts several critical aspects of liver function, including angiogenesis, the composition of the extracellular matrix, and the metabolism of drugs. These features highlight the critical need for comprehensive animal models that combine characteristics of both cirrhosis and liver cancer, to evaluate the efficacy of anticancer drugs in preventing or slowing the progression of liver cancer (2, 3).

While thioacetamide (TAA) has been used to induce liver fibrosis, cirrhosis, acute liver injury, hepatocellular carcinoma, and cholangiocarcinoma in rats through feeding or intraperitoneal injection (IP) since 1983 (4–7), its application requires long-term administration and has demonstrated a low frequency of HCC carcinogenesis. However, there have been no reports on its use in modeling early-stage, stage-specific HCC, particularly well-differentiated HCC combined with cirrhosis and with the presence of defined, aggressive cholangiocarcinomas. Diethylnitrosamine-impaired rat (DEN rat) model, though effective in simulating human hepatocarcinogenesis, does not fully replicate the entire progression from liver fibrosis to cirrhosis and eventually to HCC (8–10). To address these limitations, our study aims to develop a new rat model induced by thioacetamide (TAA), which closely mimic the progression from liver fibrosis to cirrhosis, ultimately leading to the development of HCC with the feature of early stage specific HCC (well-differentiated HCC). Utilizing this model, we intend to conduct thorough research and investigations into the effectiveness of antitumor drugs and explore the tumor microenvironment in greater depth.

## Materials and methods

### Animal models

In this research, male Lewis rats aged 4 weeks and weighing between 100-130 grams were utilized. These rats were sourced from Charles River Laboratories (MA, USA). They were housed in pairs in isolated cages within the Department of Laboratory and Animal Resources at the University of Pittsburgh. The environment for the animals was controlled for temperature and light/dark cycles. The rats had access to a standard diet and water.

We intended to create a cirrhotic liver-based HCC carcinogenesis model based on the thioacetamide (TAA)-induced liver cirrhosis model. The experimental procedure involved administering intra-peritoneal injections of TAA at a dosage of 200 mg/kg (11, 12). sourced from Sigma Chemical Co. (St. Louis, MO, USA). For the control group, saline was used. These injections were given twice weekly, with the TAA being dissolved in a saline solution at a concentration of 100mg/mL. To delineate the progression of cirrhosis and hepatocarcinogenesis induced by TAA, a total of 32 Lewis rats were subjected to a regimen of bi-weekly intra-peritoneal injections. The control group received saline injections for 34 weeks(n=16), while the experimental group was administered TAA at a dosage of 200 mg/kg for two distinct durations: 26 weeks(n=8) and 34 weeks(n=8).

Blood samples were collected from both the TAA-treated and saline-treated (control) rats at four distinct time points: prior to the start of treatment (Week 0), and at Weeks 12, 26, and 34. Tissue samples for analysis were collected at Week 34 from the control group, which received saline injections, and at Weeks 26 and 34 from the TAA-treated group.

This study adhered strictly to the Guiding Principles for the Care and Use of Laboratory Animals as established by the University of Pittsburgh. The experimental protocol received approval from the Animal Care Ethics Committee and underwent review by the local ethics committee, ensuring compliance with ethical standards in animal research.

### Chemical parameters tests

0.5mL blood samples for each rat were collected by puncture of the tail vein while they were in the fed state. Samples were analyzed by Zoetis Abaxis VetScan VS2 (Abaxis, Union City, CA) with Preventive Care Profile Plus Rotor was used to quantitatively measure the following variables: albumin(ALB), alanine aminotransferase(ALT), alkaline phosphatase(ALP), aspartate aminotransferase (AST), blood urea nitrogen(BUN), calcium(CA), chloride(CL-), creatine(CRE), globulin(GLOB), glucose (GLU), potassium (K+), sodium (NA+), total carbon dioxide(tCO2), total bilirubin(TBIL), total protein(TP). INR was analyzed by CoaguChek^®^ XS system (Roche diagnostics). AFP was analyzed by Rat αFP(Alpha-Fetoprotein) ELISA Kit (Elabscience).

### Tissue section analyses

The liver tissues were fixed in 4% paraformaldehyde (PFA) and then paraffin-embedded; four-micrometer sections of tissue were prepared. Hematoxylin-eosin (HE) staining was used for the histopathological examination. Sirius red staining according to the manufacturer’s protocols (Sigma-Aldrich) was used to detect the fibrosis development. To ascertain the HCC development, the immunohistochemical examination of localization of GST-P protein, Glypican 3 protein and Alpha- fetoprotein (AFP) were performed perceptively in liver sections. To determine whether the tumor originated from biliary epithelial cells, we performed immunofluorescence (IF) for pancytokeratin (PanCK) and immunohistochemistry (IHC) staining for Sox9. To detect vascularization, immunohistochemical examination of localization of CD34 protein and von Willebrand Factor(vWF) were performed in liver sections. To assess liver architecture to show the thickness of hepatocyte plates, the special staining of reticulin fibers (type III collagen) in the space of Disse was performed. To detect proliferating cells, immunohistochemical examination of the Ki67 was performed. For all the immunohistochemistry(IHC), 4% paraformaldehyde-fixed liver sections were applied by using the avidin-biotin complex method. Briefly, after deparaffinization (and target retrieval using Target Retrieval Citrate buffer Solution in microwave in the cases of Glypican 3, AFP, Sox9, vWF, CD34 and Ki67), the sections were treated sequentially with 3% H2O2, normal goat serum in the cases of Ki67, AFP, Sox9, vWF and GST-P or normal horse serum in the cases of CD34 and Glypican 3, primary antibody (i.e., mouse anti-rat monoclonal IgG1 Glypican 3(,1:200, room temperature, 1 hour, ab216606/Abcam), rabbit anti-rat polyclonal IgG AFP(1:250, room temperature, 1 hour, PA5-21004/Invitrogen), rabbit anti-rat polyclonal IgG Sox9(1:200, room temperature, 1 hour, ab5535/Sigma-Aldrich), rabbit anti-human polyclonal von Willebrand factor (1:200, room temperature, 1 hour, A0082/Agilent Dako), rabbit anti-rat monoclonal IgG CD34(1:100, room temperature, 1 hour, ab81289/abcam), mouse anti-rat monoclonal IgG Ki67(1:50, room temperature, 1 hour, 550609/BD) and rabbit anti-rat GST-P polyclonal antibody (ready to use, room temperature, 1 hour; 311-H/MBL), biotin-labeled goat anti-rabbit IgG or biotin-labeled horse anti-mouse IgG and avidin-biotin-peroxidase complex (VECTASTAIN Elite ABC HRP kit; Vector Laboratories PK6101). Counterstaining by hematoxylin for 30 seconds. Images were captured by using the Olympus IX71 inverted microscope (Olympus, Tokyo, Japan) and collected by cellSens Dimension software. The positive area threshold was quantified using ImageJ software (NIH, Bethesda, MD, USA).

For immunofluorescence (IF) for pancytokeratin (PanCK), after deparaffinization (and target retrieval using Target Retrieval Citrate buffer Solution in microwave), the sections were treated sequentially with Sodium Borohydride(1mg/mL), normal donkey serum, primary antibody(mouse anti-pan Cytokeratin, 1:200, room temperature, 1 hour, ab7753/Abcam), secondary antibody(Alexa FluorTM 594 donkey anti-mouse IgG, 1:250, A21203/Invitrogen) 1h room temperature. Sections were covered with mounting media with DAPI. Images were captured using the same microscope as previously described.

### Statistical analysis

All the data were tested for normality and the appropriate statistical test was chosen. The comparisons of means were calculated by using ANOVA tests with Tukey HSD correction for multiple means comparisons, and independent T-tests only when two means were compared. The data are presented as mean values _ standard error mean (SEM). The statistical analyses were performed using Prism 10 (GraphPad Software Inc., San Diego, CA, USA).

## Result

### Characterization of chronic TAA-induced liver cirrhosis

**Figure 1:**
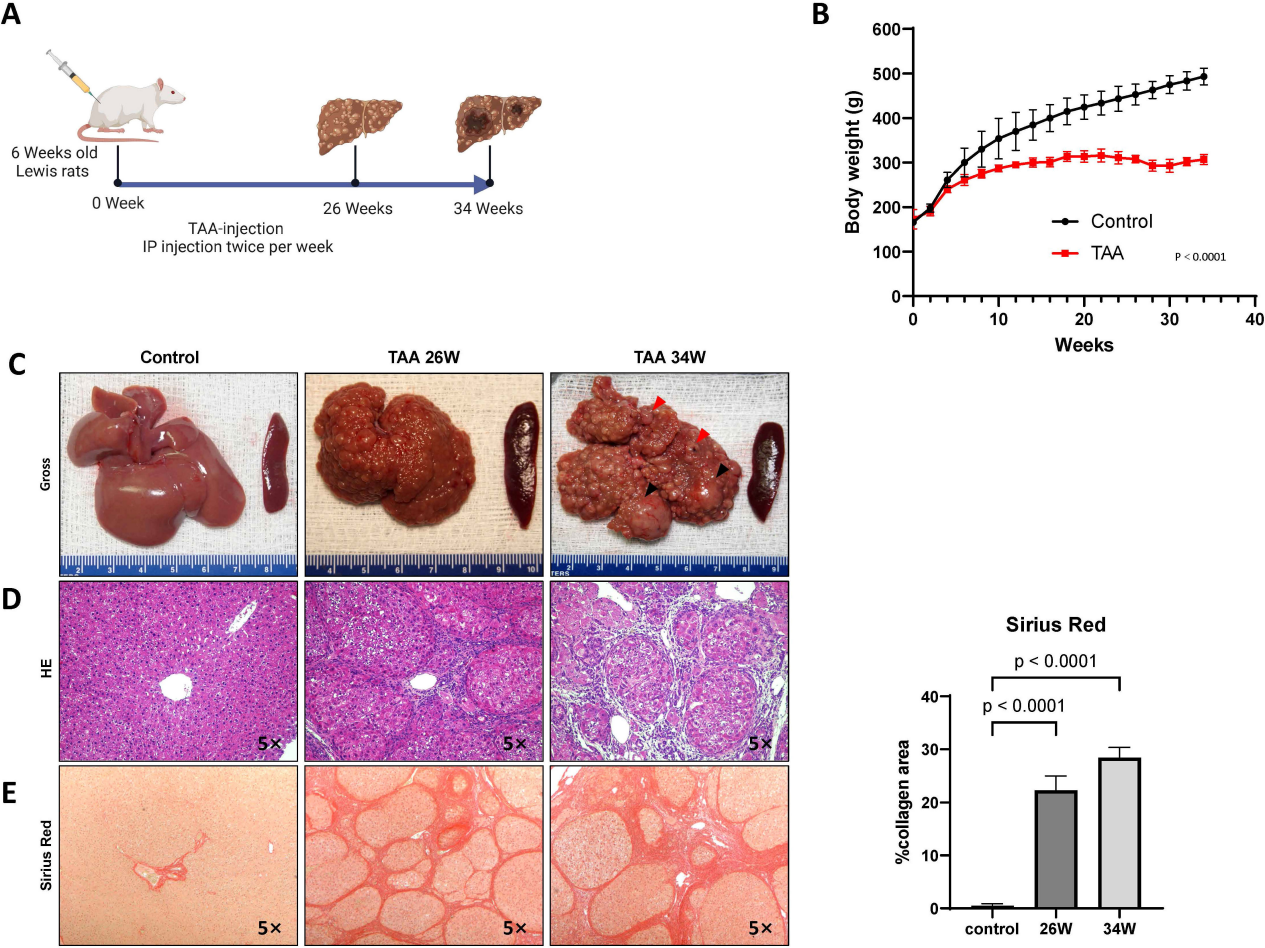
Chronic TAA-induced cirrhosis. **(A)** Timeline of the protocol for the TAA-induced cirrhotic rat model of HCC. Six-week-old Lewis rats received bi-weekly injections (200 mg/kg). **(B)** Graphical representation of rat body weight change in both TAA injection group and saline injection group during treatment. The x-axis represents the treatment duration rather than rat age. Statistics evaluation was performed via two-way ANOVA. p<0.0001, n=15 (Control group), n=15 (TAA group). Error bars are ± STD. **(C)** Gross photographic documentation of rat liver morphology and histological staining images (H&E **(D)**, Sirius Red **(E)**) at 26, 34 weeks after TAA treatment. Saline injection group used as control. Histological sections were obtained from rat tissue using paraffin embedding techniques. Magnification 5x. Graph on right representing Sirius staining quantification in E by identifying Sirius red stained area per high power field (HPF). Statistic analysis was performed by two-way ANOVA following Šídák’s multiple comparisons test between control group and two time points in TAA group. Sirius red stained area in two TAA group is significantly higher than control group (Both p<0.0001, n=5). Bars show mean ± STD.

Following our scheme (**Figure 1A**), TAA treatment inhibited body weight gain in the rats (**Figure 1B**) and induced progressive liver damage, culminating in the development of cirrhotic nodules in 100% of the TAA-treated animals after 26 weeks of injections (**Figures 1C, D**). As summarized in **Table 1**, ductular proliferation was apparent as early as after 22 weeks of TAA administration which expanded after 26 weeks and transitioned to malignant cholangiocarcinomas with extensive atypical bile ducts. By the 34-week time point, obvious invasive cholangiocarcinomas were identified throughout the liver. Regarding hepatocyte neoplasia, by 22 weeks, we observe evidence of possible clonality in cirrhotic nodules and by 26 weeks cirrhotic nodules look more “chord-like” which is typical of HCC in addition to obvious evidence of HCC cells, as verified by our clinical pathologist, in 25% of rats at this time point invading into the surrounding connective tissue. All rats that continue with TAA administration to the 34-week mark develop massive HCC nodules together with defined cholangiocarcinoma demonstrating for the first time that TAA administration under the right conditions can generate a mixed liver cancer phenotype. We assessed overall fibrosis using Sirius red staining. This histological analysis revealed a significant increase in fibrosis in the TAA-treated rats at both the 26-week (p = 0.0009) and 34-week (p < 0.0001) time points, compared to the control group (ANOVA, p < 0.0001) (**Figure 1E**). These results demonstrate the efficacy of TAA in inducing liver cirrhosis in a controlled experimental setting, providing a valuable model for studying the pathophysiological mechanisms underlying liver disease progression.

**Table 1:** Pathological features of TAA induced hepatic neoplasia.

Time Point (weeks)	Fibrosis	Presence of Isolated Cirrhotic Nodules	Presence of Nodule Clonality	Ductular Proliferation	Presence of CCA	Presence of HCC
22	+	+	+	++	+	–
26	++	++	+++	+++	++	+
34	++	++	+++	++++	++++	+++

-, absence/no detection; +, minimal/mild presence; ++, moderate presence; +++, high presence; ++++, severe presence.

### Phenotypic characterization of chronic TAA-induced hepatocarcinogenesis HCC

**Figure 2:**
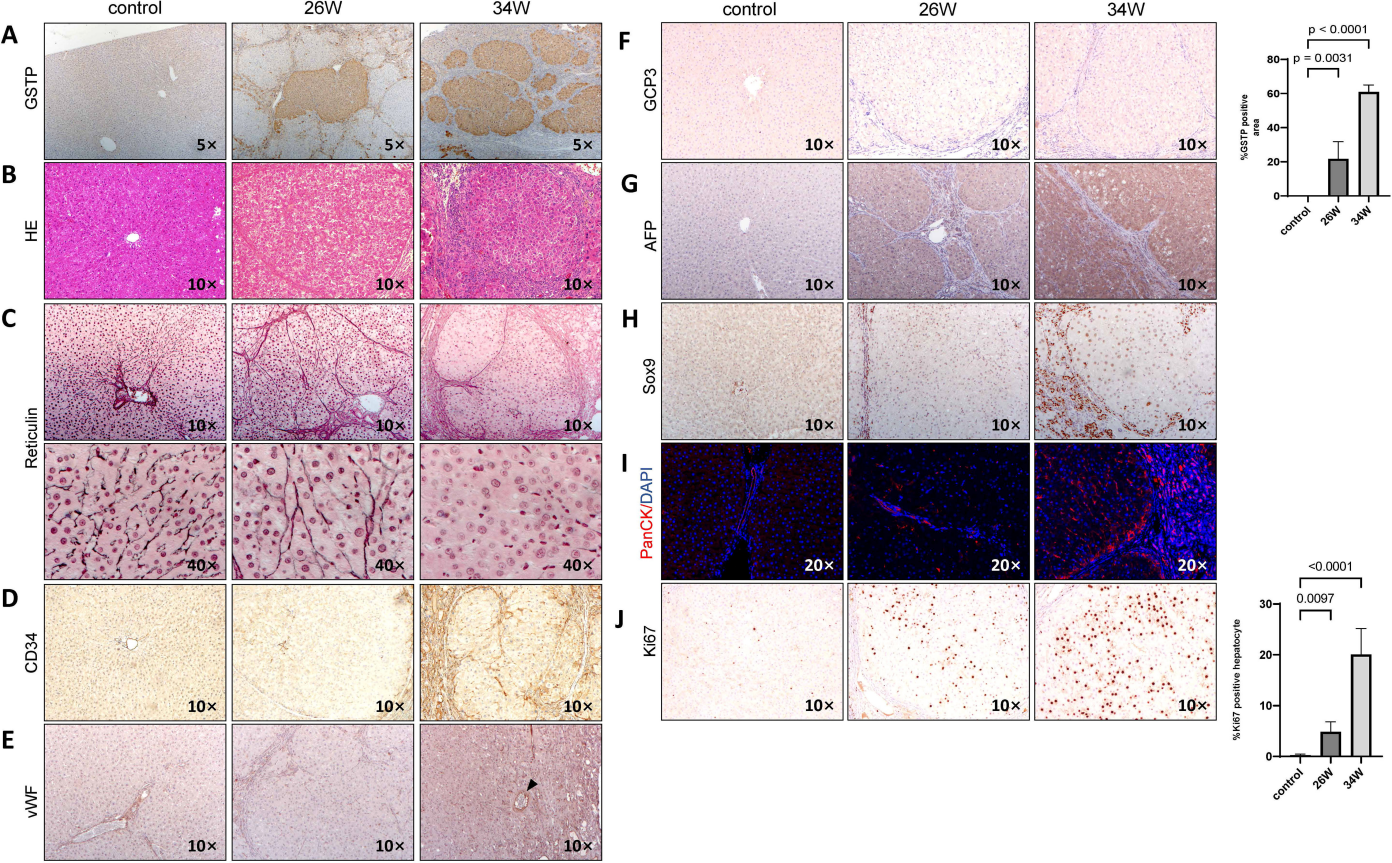
Chronic TAA-induced hepatocarcinogenesis. Photographic documentation of rat liver histological staining images and immunohistochemistry (IHC) staining images at 26 and 34 weeks after TAA treatment. Saline injection group used as control. Rat liver sections were obtained using paraffin embedding techniques. **(A)** GST-P IHC staining, with GST-P positive quantification of GST-P positive nodule surface area per HPF on the right side. Statistics analysis was performed via two-way ANOVA following Šídák’s multiple comparisons test between control group and two time points in TAA group. GST-P positive rate in two TAA group is significantly higher compared to control group(26W: p=0.0031, n=5, 34W: p<0.0001, n=5). Magnification 5x. **(B)** H&E staining, magnification 10x. **(C)** Reticulin staining, magnification 10x and 40x. **(D)** CD34 IHC staining, magnification 10x. **(E)** vWF IHC staining. magnification 10x. Black arrows point to isolated artery. **(F)** GCP3 IHC staining, magnification 10x. **(G)** AFP IHC staining, magnification 10x. **(H)** Sox9 IHC staining, magnification 10x. **(I)** PanCK IF staining, magnification 10x. **(J)** Ki67 IHC staining, with quantification of Ki67 positive nodule numbers and surface area per HPF on the right side. Statistic analysis method same with A, Ki67 positive rate in two TAA group is significantly higher compared to control group(26W: p=0.0097, n=5, 34W: p<0.0001, n=5). Bars show mean ± STD.

At 34 weeks, rat livers exhibited nodules of varying sizes, with fused tumor nodules measuring 0.5-0.7 cm(black arrows in **Figure 1C**) and a large number of small tumor nodules measuring 0.1-0.3 cm(red arrows in **Figure 1C**).To investigate the hepatocarcinogenic effects of chronic TAA administration, we focused on cellular differentiation and proliferation. The presence of GST-P positive lesions, indicative of preneoplastic and neoplastic changes, is significantly evident following 26 weeks of TAA treatment (p < 0.0001), with a notable expansion observed at 34 weeks (p < 0.0001) (**Figure 2A**). Hematoxylin and eosin (HE) staining of GST-P positive lesions in the liver tissues at 26 weeks post-TAA treatment revealed characteristics of cirrhotic structures. By 34 weeks, the lesions exhibited features typical of well-differentiated HCC and cholangiocarcinoma, including enriched hepatocyte cytoplasm, increased nucleoplasm ratio, expanded hepatocyte plates, and invasive ductular reaction with bile duct proliferation (**Figure 2B**). Further histological analysis involved reticulin and CD34 staining to assess liver architecture and vascular organization. In control rats, the reticulin network is well-preserved. In contrast, TAA-treated rats at 26 weeks displayed a normal reticulin pattern with thick expression along the liver sinusoids and hepatic trabeculae, involving fewer than three cell layers. However, by 34 weeks of TAA treatment, a marked disappearance and collapse of the reticulin network is observed in neoplastic regions (**Figure 2C**). Additionally, CD34 staining at 34 weeks post-TAA treatment showed diffused positivity compared to controls and the 26-week TAA group (**Figure 2D**). vWF staining demonstrated tumors at 34 weeks post-TAA treatment are supplied blood by isolated arteries (indicated by the black arrow in **Figure 2E**). Glypican-3 staining, which is associated with advanced HCC stages (13), is negative in the lesions at 34 weeks of TAA treatment (**Figure 2F**). AFP staining is positive in the tissue section of 34 weeks of TAA treatment assisted pathological diagnosis of HCC (**Figure 2G**). All the support suggesting a well-differentiated state of HCC. These findings align with established criteria for diagnosing well-differentiated HCC, where the absence or reduction of reticulin stain and abnormal reticulin patterns with widened trabeculae are considered reliable indicators (14).

For checking the tumor phenotype, Sox9 and PanCK staining showed small, atypical bile duct proliferation in the 26-week TAA treatment group, but more pronounced ductular reaction and bile duct proliferation at 34 weeks which is indicative of cholangiocarcinoma (**Figures 2H, I**).

When measuring hepatocyte proliferation, a marked increase in Ki67 positive nuclei is observed at 34 weeks of TAA treatment compared to both the control group and the 26-week TAA group (p < 0.0001) (**Figure 2J**). At 34 weeks of TAA treatment, the Ki67 expression in peri-tumoral regions is lower than that in tumor tissues (**Supplementary Figure S1**). These results collectively indicate that chronic TAA administration leads to the development of well-differentiated HCC, characterized by distinct histopathological changes, increased hepatocyte proliferation, and partial biliary characteristics.

### Biochemical profile of animals under chronic TAA-induced hepatocarcinogenesis in cirrhotic liver

**Figure 3:**
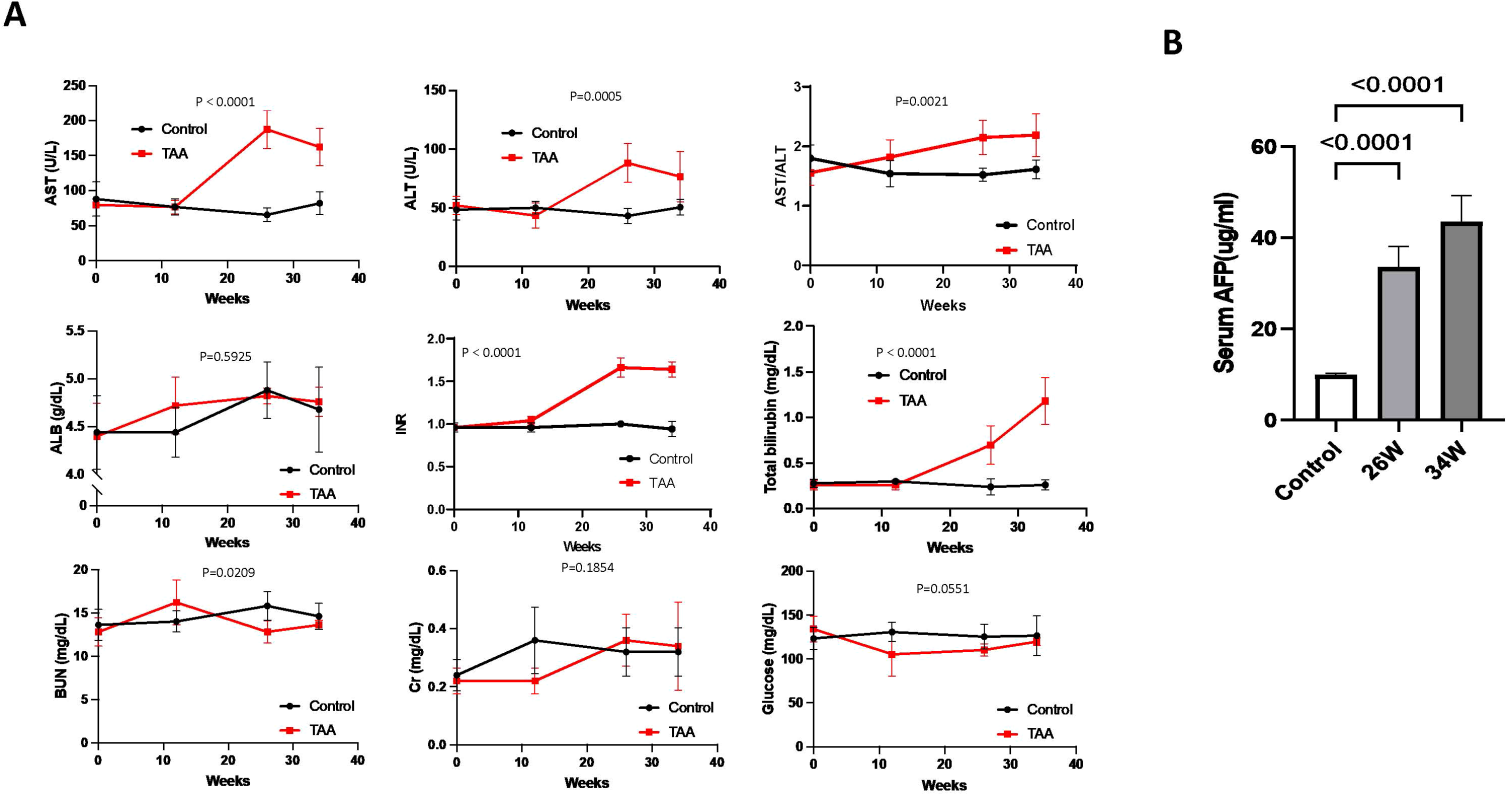
Biochemical markers, body weight changes, and serum AFP levels during chronic TAA-induced hepatocarcinogenesis. **(A)** Graphic representation of variations in clinically-relevant serum parameters for rats in both the TAA injection group and the saline injection control group during treatment, p values showed in graph. Statistics analyses were performed using two-way ANOVA. Post-TAA treatment, a significant increase in AST, ALT, AST/ALT ratio, PT/INR and total bilirubin level was observed while other parameters tested remains no significant change. Error bars show mean ± STD. **(B)** Graphic representation of serum AFP levels in control, 26-week, and 34-week TAA-treated groups by ELISA. Rat serum was isolated from fresh blood samples by centrifugation. Statistics analysis was performed using two-way ANOVA following Šídák’s multiple comparisons test between control group and two time points in TAA group. Post-TAA treatment, a significant increase in serum AFP level was observed (both p<0.0001, n=8). Bars show mean ± STD.

Biochemical analysis revealed notable changes in the TAA-induced hepatocarcinogenesis model. Specifically, levels of ALT, AST, the ALT/AST ratio, TBIL, and INR are significantly elevated in the TAA group compared to the control group at both week 26 and week 34 (**Figure 3A**). In contrast, levels of ALB, BUN, Cr, and glucose did not show any significant difference between the TAA-treated group and the control group (**Figure 3A**). Additionally, the level of serum AFP, a marker often associated with liver cancer, was found to be higher in the TAA group at both week 26 and week 34 compared to the control group (**Figure 3B**). This increase in AFP levels further corroborates the development of hepatocarcinogenesis in the TAA-treated rats.

Based on these results, a thioacetamide-induced cirrhosis combined with mixed HCC rat model is established, exhibiting HCC pathological characteristics such as hepatocyte proliferation, liver fibrosis/cirrhosis, widened hepatocyte plates, disorganized vasculature and bile duct proliferation. Additionally, our model displays parameters of chronic liver function impairment, thereby replicating the typical processes observed in the progression of human HCC.

## Discussion

The significant association of HCC with chronic liver damage, leading to fibrosis or cirrhosis, is underscored by the fact that approximately 90% of HCC cases arise from these conditions. Liver fibrosis or cirrhosis plays a crucial role in the malignant transformation of liver tissue, as highlighted in various studies (15). The changes induced by these conditions, such as altered liver vascular formation, modifications in the extracellular matrix composition, and impacts on drug metabolism, emphasize the necessity of employing animal models that effectively combine features of both cirrhosis and liver cancer. These models are instrumental in investigating the mechanisms through which anti-tumor drugs influence tumor initiation and development (4, 16, 17).

Thioacetamide (TAA) is a well-established chemical for inducing liver fibrosis, acute liver injury, and models of hepatocarcinogenesis in rats and mice (4–6). Historically, modeling of hepatocarcinogenesis with TAA required long-term administration and demonstrated low hepatocellular carcinoma carcinogenic efficiency (7). Additionally, TAA alone administered orally tend to more efficiently induce cholangiocarcinoma (18). With the advent of diethylnitrosamine (DEN), which more efficiently promotes HCC carcinogenesis, most models now use DEN, sometimes in combination with TAA (19). For TAA dosage and frequency of administration, IP dosages ranged from 200 to 400 mg/kg, administered 2 to 3 times per week or every other day, depending on the specific model setup. To model liver cancer formation based on chronic liver injury, we opted for a relatively lower dose of 200 mg/kg, administered twice a week (11, 12). Combining DEN with TAA is commonly used to promote hepatic disease in a relatively short time injection, however it does not completely mimic the progression from liver fibrosis to cirrhosis and subsequent HCC development (6, 8, 9). Nor, have any other group reported that DEN/TAA combination results in mixed liver cancer phenotype. Most studies utilizing TAA alone either pursue the investigation of fibrosis/cirrhosis, or promote the development of cholangiocarcinoma. HCCs in the background of TAA administration usually occur with multiple confounding factors such as hepatic steatosis, or with other chemical agents (20). In response to this gap, our study developed a TAA-induced rat model that effectively combines cirrhosis with HCC, closely mirroring the progression observed in human HCC. This model, through specific staining and histological examination, predominantly exhibits well-differentiated HCC as well as cholangiocarcinomas, aligning with the increasing prevalence of early-stage liver cancers in clinical settings.

Biochemically, the TAA-induced cirrhosis and HCC model demonstrated significant elevations in AFP levels, a key tumor marker for HCC, suggesting the onset of liver carcinogenesis (20–22). Glypican-3, common diagnostic marker for hepatocellular carcinomas in human, is negative for our HCC model. Glypican-3 expression tends to increase in more advanced HCC pathologies and our model more replicates HCC at early stages in rats (23). Partially positivity for SOX9 and PanCK in HCC could suggest the tumor may exhibit features of stemness to hepatocellular and biliary differentiation which falls in line with the mixed phenotype we observe (24). Other indicators, such as increased INR, elevated TBIL levels, high AST and relatively lower ALT levels, pointed towards abnormal liver function. Notably, the AST/ALT ratio greater than 2, typically seen in alcoholic liver cirrhosis, was also observed in our model, suggesting potential mitochondrial damage caused by TAA metabolites (25). The elevated INR is indicative of impaired liver synthetic function, the increased TBIL could be attributed to bilirubin metabolism dysfunction. However, the maintenance of normal albumin and blood sugar levels, along with the absence of ascites and hepatic encephalopathy, indicated that liver impairment was not end-stage. Additionally, normal kidney function suggested that TAA did not induce renal damage. Compared to other rodent models of HCC (2, 5, 8, 9), our model demonstrated liver function impairment without affecting other organs, thereby more closely resembling the clinical characteristics of HCC.

Nevertheless, this model possesses certain limitations. To achieve a 100% incidence rate of HCC, a prolonged period exceeding 34 weeks was necessary. It is also known that like other carcinogen-induced models, TAA promotes development of tumor lesions with certain mutational landscape. This differs from what is seen in clinical practice, where high level of case-to-case, lesion-to-lesion or even intertumoral heterogeneity is observed. Furthermore, additional research is required to elucidate the molecular mechanisms underpinning hepatocarcinogenesis associated with TAA-induced cirrhosis.

In conclusion, the establishment of a rat model with TAA-induced cirrhosis and HCC in this study offers a comprehensive understanding of the pathological characteristics and progression of early-stage liver cancer with concurrent liver function impairment. This model provides a more accurate reflection of clinical features than other animal models and is crucial for future research into the mechanisms of anti-tumor drugs in tumor initiation and progression.

## Data Availability

The raw data supporting the conclusions of this article will be made available by the authors, without undue reservation.
