# Supplementary material for: A rat model of cirrhosis with well-differentiated hepatocellular carcinoma induced by thioacetamide
Source: Front Gastroenterol (Lausanne). 2024 Nov 12;3:1427820. doi: 10.3389/fgstr.2024.1427820 (PMC12952341; doi:10.3389/fgstr.2024.1427820)
Supplement: Supplementary file 1 [file DataSheet1.pdf]

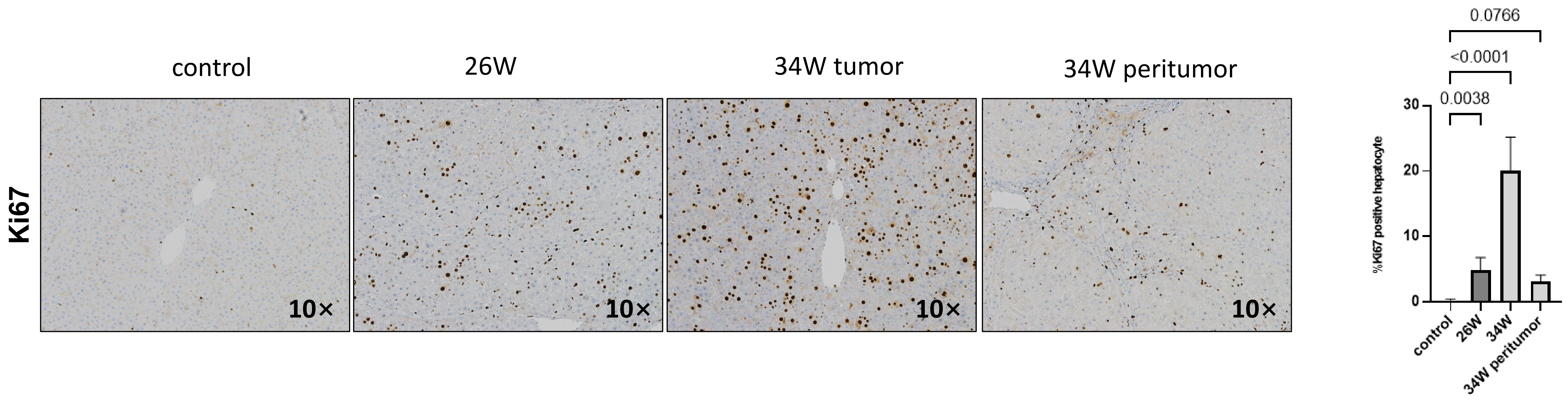

Supplement Figure 1: Ki67 assessment of neoplastic regions during TAA administration

Immunohistochemical analysis was performed to detect the expression levels of Ki67 in the control group, 26-week TAA group tissues, 34-week TAA group tumor tissues, and peritumoral tissues. Liver samples were selected from five rats in each group, and tissue sections were prepared for Ki67 immunohistochemistry (IHC) staining. For each section, images were captured from three different fields of view. As noted, the intra-tumoral and peri-tumoral regions were quantified at the 34W time point where there was evidence of clear HCC and cholangiocarcinoma. The number of Ki67-positive cells was quantified using ImageJ software, and the average value from the three fields was calculated for further analysis. Data are represented as mean ± SD.
